# Marginal bone loss in relation to platform switching implant insertion depth: An update

**DOI:** 10.4317/jced.50743

**Published:** 2012-07-01

**Authors:** Rocío Alonso-González, Amparo Aloy-Prósper, David Peñarrocha-Oltra, M A. Peñarrocha-Diago, M. Peñarrocha-Diago

**Affiliations:** 1 DDS. Resident of the Master in Oral Surgery and Implantology. Valencia University Medical and Dental School.; 2 DDS. Master in Oral Surgery and Implantology. Valencia University Medical and Dental School.; 3 Associate Professor of Oral Surgery. Valencia University Medical and Dental School.; 4 Chairman of Oral Surgery. Director of the Master in Oral Surgery and Implantology. Valencia University Medical and Dental School. Valencia (Spain).

## Abstract

A review is made, analyzing marginal bone loss in relation to the depth of implant insertion with platform switching, according to the position of the neck (supracrestal, crestal or subcrestal), and evaluating survival of the implants. A PubMed search was made of the studies in animals and humans published between 2005 and 2011, specifying platform insertion depth (supracrestal, crestal or subcrestal) and registering marginal bone loss from the time of prosthetic restoration to the end of follow-up (minimum 6 months). A total of 30 studies were included. The bone loss associated with implants placed at supracrestal level was slightly smaller than in the case of implants placed at subcrestal level, though statistical significance was not reached. The mean marginal bone loss values were 0.0 mm to 0.9±0.4 mm for the implants with the neck located at supracrestal level; 0.05 mm to 1.40±0.50 mm for those at subcrestal level; and 0.26±0.22 mm to 1.8±0.39 mm for those in a crestal location, after 6-60 months of follow-up. The survival rate was 88.6-100% for the implants with the neck positioned at crestal level, versus 98.3-100% below the crest, and 100% above the crest. The heterogeneity of the studies (surgical technique, platform surface texture, radiographic measurement techniques, etc.) made it difficult to establish a relationship between marginal bone loss and the supracrestal, crestal or subcrestal location of platform switching.

** Key words:**Dental implants, platform switching, insertion depth, crestal insertion level, bone loss.

## Introduction

The aim of dental implantology is to preserve the peri-implant tissues over the long term. However, marginal bone loss has been described in the early stages following prosthetic restoration, in apparent relation to the location of the implant-abutment interface. Factors such as bacterial infiltration (1,2), micro-movements (2) and the transmission of stress at the implant-abutment interface give rise to apical migration of the biological width (3) in order to isolate and protect the bone from irritation. With platform switching, the implant-abutment interface (IAI) is displaced horizontally towards the center of the platform and separated from the marginal bone. Thus, bacterial infiltration, micro-movements and stress occur at a distance from the marginal bone, giving rise to lesser apical migration of the biological width (2) and therefore to less marginal bone reabsorption. Such bone loss is also conditioned by the implant platform insertion depth, as specified according to the vertical location of the implant-abutment interface (i.e., supracrestal, crestal or subcrestal).

Marginal bone loss of implants with platform switching has been related to the length (2,4), diameter (2) and surface of the neck (3,5), and to the inter-implant distance (6). Regarding insertion depth, some authors recom-mend placing the platform at crestal level or 1-2 mm below the crest (3,7), with the argument that this results in improved maintenance of the marginal bone (8). However, the coexistence of other factors (surgical technique, platform surface texture, radiographic measurement techniques, etc.) makes it difficult to establish a direct relationship between marginal bone loss and the supracrestal, crestal or subcrestal location of platform switching.

The present review analyzes marginal bone loss in relation to the depth of implant insertion with platform switching, according to the position of the neck (supracrestal, crestal or subcrestal), and evaluates survival of the implants.

## Inclusion criteria and search strategy

A PubMed review was made, covering the period between January 2005 and April 2011, referred to marginal bone loss in implants with platform switching. The search included animal and human clinical and/or histological studies specifying platform insertion depth (supracrestal, crestal or subcrestal) and registering the level of bone loss from the time of prosthetic restoration to the end of follow-up (minimum 6 and 12 months for the animal and human studies, respectively. We excluded those studies using bone augmentation techniques and those involving heavy smokers (over 20 cigarettes/day), since the baseline clinical conditions would be different, and the results therefore would not be comparable. A total of 30 studies were included.

The PubMed search was based on the following keywords: platform switching and dental implants, crestal level placement, installation depth, platform switching and crestal level, platform switching and bone loss. Articles from the following journals were included: International Journal of Oral and Maxillofacial Implants, Journal of Oral and Maxillofacial Surgery, International Journal of Periodontics and Restorative Dentistry, International Journal of Prosthodontics, Clinical Oral Restorative, and Medicina Oral Patologia Oral y Cirugia Bucal.

A total of 105 articles were identified, of which 75 were excluded: 12 finite elements analyses; 10 articles repor-ting no data on bone loss from prosthetic restoration to the end of follow-up; 7 studies involving placement with bone grafts; one article with a follow-up of under 6 months; one study involving smokers of over 20 cigaret-tes/day; and 44 articles that failed to specify the level of insertion of the implant platform, or which could not be accessed. A total of 30 studies were thus finally included. The following data were collected from the most relevant publications: authors and year of publication, type of study, the presence of a control group (conventional platform), the number of implants, the diameters of the platforms and abutments, platform insertion depth, marginal bone loss, duration of follow-up, and implant survival rate. The following confounding factors were also registered: implant system (manufacturer), treatment of the implant neck (smooth/rough), and a surgical technique in one or two steps (submerged or non-submerged)( Table 1,  Table 2).

**Table 1:**
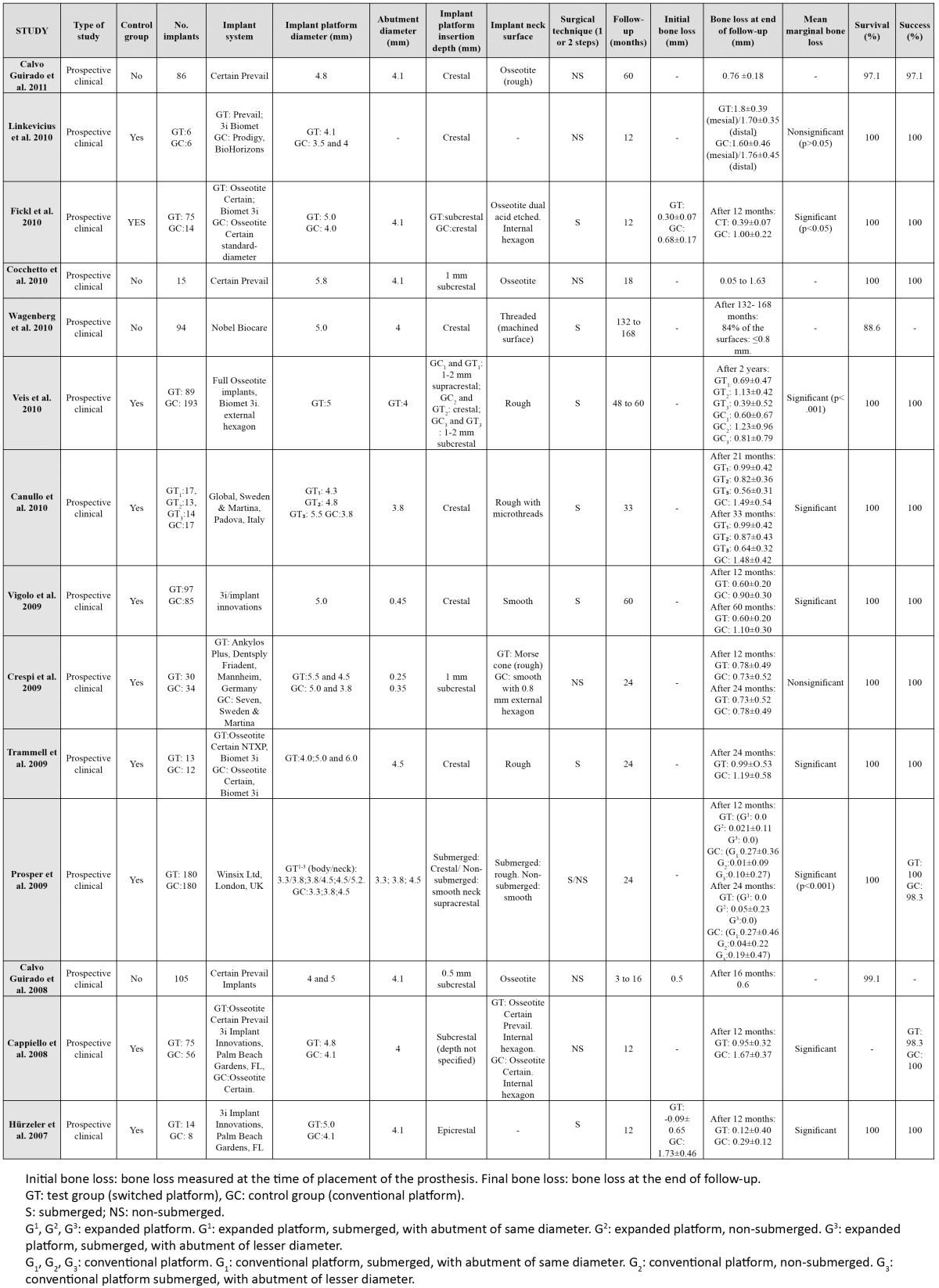
Human clinical studies with / without a control group.

**Table 2:**
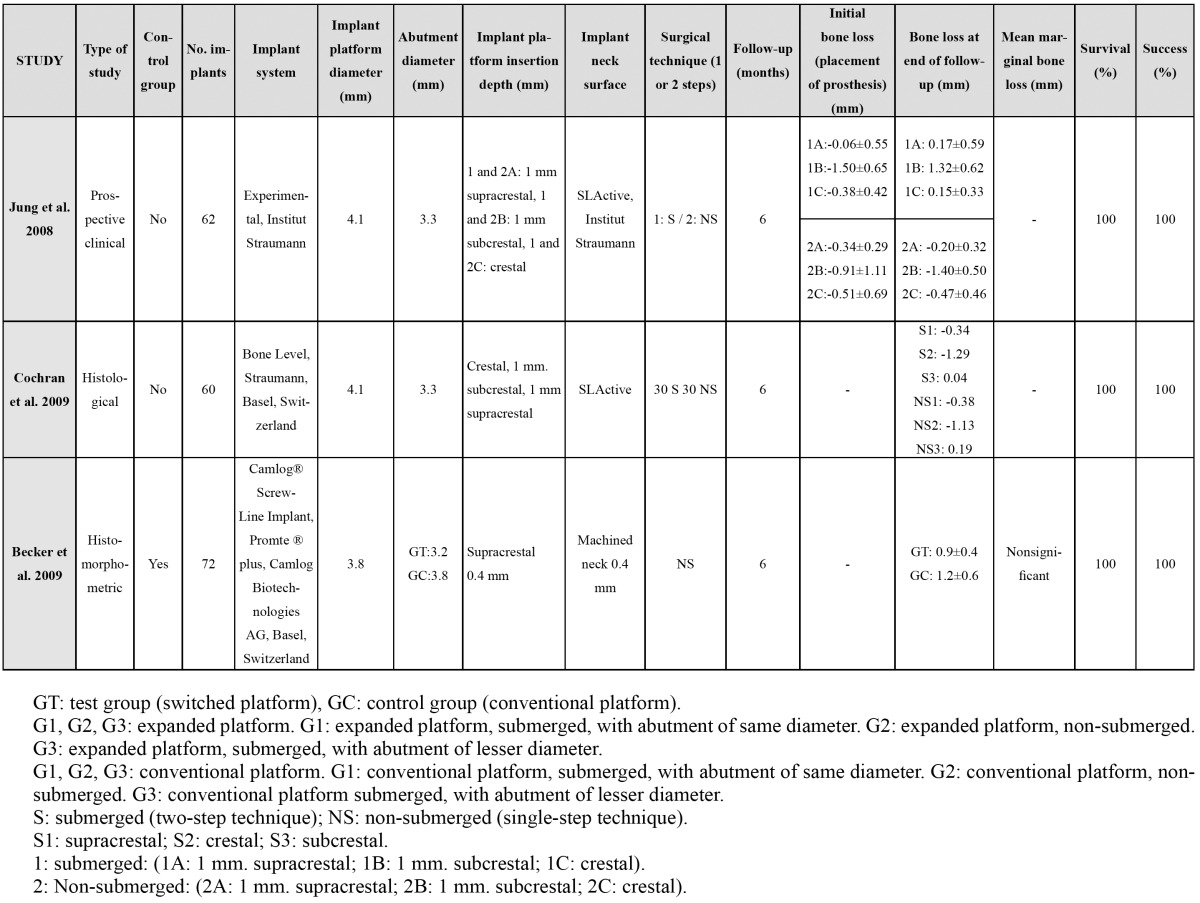
Animal studies with / without a control group.

## Results

Marginal bone loss according to platform insertion depth

Peri-implant marginal bone loss is conditioned by the position of the implant-abutment interface (IAI) in the bone crest, horizontally and vertically (9).

With platform switching, the IAI is displaced horizontally towards the center of the platform and separated from the marginal bone. Thus, bacterial infiltration, micro-movements and stress occur at a distance from the marginal bone, giving rise to lesser apical migration of the biological width (2) and therefore to less marginal bone reabsorption. Most comparative studies in humans (2,4,7,8,10-13) and animals (14) have reported greater marginal bone loss with conventional platforms than with platform switching, though some authors (15-17) have found no significant differences.

The IAI is associated with a peri-implant inflammatory infiltrate that is partly responsible for crestal bone reabsorption (18). A recent study in dogs (1) has shown the magnitude of the peri-implant inflammatory infiltrate to depend on the insertion depth of the IAI with respect to the alveolar crest (supracrestal, crestal or subcrestal). Few studies in the literature have evaluated the influence of the insertion depth of implants with platform swit-ching with respect to bone loss (3,10,14,18).

Crestal position of the platform

A crestal position is defined when the most coronal portion of the implant platform is positioned at the level of the crestal bone inter-proximally (19).

In different human studies (2,4,11,12) the implant platforms were inserted at bone crest level. In the studies included in the present review, bone loss for the platforms positioned at crestal level varied from 0.26±0.22 mm (18) to 1.8±0.39 mm (16), after a follow-up ranging from 6 months (14,17,18) to 60 months (10,11,20)(Table 1, Table 2).

Vela-Nebot et al. (21) recorded a mean bone loss of 0.76 mm and 0.77 mm mesial and distal, respectively, after 12 months of follow-up. Accordingly, they recommended the use of platform switching at crestal level for preservation of the marginal bone level. When the IAI is located at crestal or subcrestal level, the reabsorption of 2 mm of marginal crestal bone is observed as a result of establishment of the biological width, which acts as a mucosal barrier over the crestal bone (10). According to Wagenberg et al. (19), the amount of bone reabsorption required to establish the biological width decreases when the implant platform is placed at crestal level. These authors observed a marginal reabsorption of ? 0.8 mm at 84% of the measured surfaces, 11-14 years after restoration. In this sense, a study in dogs (14) recorded minimum bone loss when the platforms were placed at crestal level (0.34 mm) and subjected to loading during 6 months.

However, in a comparative study involving 24 months of follow-up, Veis et al. (10) recorded greater marginal bone reabsorption with the crestal position than with subcrestal insertion, for both conventional platforms and platform switching. The bone loss values for conventional platforms and platform switching at crestal level were 1.23±0.96 and 1.13±0.42 mm, respectively.

Supracrestal position of the platform

According to the studies evaluated in the present review, bone loss with platforms placed at supracrestal level varied from 0.0 mm (4) to 0.9±0.4 mm (17), after a follow-up period of between 6 months (14,17,18) and 60 months (10,11,20) (Table 1, Table 2).

Hürzeler et al. (13) inserted 14 platform switching implants at supracrestal level, without specifying the milli-meters of depth. The associated bone loss was 0.22±0.53 mm after 12 months of prosthetic loading. Veis et al. (10) in turn placed 34 platform switching implants at supracrestal level, with a recorded bone loss of 0.69±0.47 mm after 24 months of follow-up.

In a histological study in dogs, Becker et al. (17) placed the platforms 0.4 mm above the bone crest. After one month of oral exposure, the distance between the shoulder of the implant and the first bone-implant contact point (fBIC) increased slightly and then remained stable over the next 5 months. These authors postulated that horizontal disadjustment in platform switching could help reduce the vertical dimension of the biological width. However, according to the authors, this was not enough to avoid marginal bone reabsorption, though the values were low, with an average of 0.9±0.4 mm after 6 months of follow-up. Histologically, the authors observed inflammatory infiltration in the proximity of the implant-abutment interface. Although the implant platform was positioned above the bone crest and therefore away from the bone, it was not possible to estimate the degree to which the inflammatory infiltrate could have influenced marginal bone loss during the early stages. Based on these results, platform switching could have a limited effect in the prevention of marginal bone reabsorption. According to Veis et al. (10), these results were due to the supracrestal position of the platform.

Several authors have suggested an association between bone loss and platform insertion depth (1,14), with in-flammatory infiltration as a linking factor: one way to separate inflammation from the bone is to vertically displace the implant-abutment interface with respect to the bone crest, i.e., placing the platform at supracrestal level. In this sense, Cochran et al. (14) reported that lesser bone losses would be obtained with platforms placed at supracrestal level. These authors placed 12 switched platforms at three possible depths (crestal, 1 mm subcrestal, and 1 mm supracrestal), obtaining a gain of 0.04 to 0.19 mm in marginal bone with the platforms placed 1 mm above crestal level. In this same line, Jung et al. (18) recorded lesser marginal bone loss with the platforms placed at crestal level and 1 mm above crestal level.

According to Hermann et al. (22), if the implant-abutment interface (IAI) is positioned above crestal level, marginal bone loss will be smaller than when positioned below crestal level, because the supracrestal position increases the distance between the inflammatory infiltrate at the IAI and the crestal bone.

Subcrestal position of the platform

Among the studies considered in our review, the bone loss recorded with platforms placed at subcrestal level ranged from 0.05 mm (23) to 1.40±0.50 mm (18) after a follow-up of between 6 months (14,17,18) and 60 months (10,11,20) ( Table 1,  Table 2).

According to Hermann et al. (22), placing the implant-abutment interface at subcrestal level can cause vertical bone reabsorption to establish the biological width. In some studies (1,22), when the IAI was positioned below crestal level, the inflammatory infiltrate was found to be greater, and the resulting vertical bone reabsorption increased. Cochran et al. (14) and Jung et al. (18) also recorded greater marginal bone loss with platforms inserted 1 mm subcrestal, in both submerged and non-submerged implants (1.29 and 1.13 mm; and 1.32 and 1.40 mm, respectively); however, these values included 1 mm corresponding to insertion depth with respect to the crest. Therefore, minimum bone loss occurred around the subcrestal platform (14).

Other studies contradict the above findings. Degidi et al. (9) inserted the implant platform 2 mm subcrestal. After one year of prosthetic loading there was no marginal bone loss, and the crestal bone was maintained 2 mm above the implant platform. According to Veis et al. (10), platform switching is only of benefit when positioned below crestal level. These authors inserted 89 switched platforms at all levels (1-2 mm supracrestal, crestal and 1-2 mm subcrestal); subcrestal platform switching yielded the lowest bone reabsorption values, followed by supracrestal placement (0.39±0.52 mm and 0.69±0.47 mm, respectively) after 24 months of prosthetic loading. The largest bone loss values corresponded to insertion at crestal level (1.13±0.42 mm). According to Veis et al. (10), the aforementioned studies (1,18) recorded greater bone loss with the subcrestal platforms because of the radiographic measurement technique used: the authors measured the distance between the bone crest and the first bone-implant contact point (fBIC). Veis et al. (10) used the implant-abutment interface and fBIC as reference points.

Lee et al. (24) inserted 308 platform switching implants with hydroxyapatite (HA) coated necks and 305 titanium plasma-sprayed (TPS) implants. Some were placed at crestal level, and others 2 mm subcrestal. The HA implants placed at crestal level failed 2.89 times more often than those positioned 2 mm below crestal level. This appears to show that when the rough surface is exposed to the oral environment, bacterial contamination and greater bone loss can be expected. In the case of platforms positioned at subcrestal level, the mucosal barrier increases while bone loss decreases (25).

Confounding factors

The different implant designs and geometries, among other factors, can influence bone remodeling after implant placement (18), independently of the level of insertion of the platform.

The texture of the implant neck surface (smooth or rough) plays an important role in relation to marginal bone loss (24,26). Vais et al. (10) took this into account by using platforms all with the same roughness, in order to avoid this confounding factor. In the study published by Prosper et al. (4), the neck of all the submerged implants had a rough surface, while the non-submerged implants presented a smooth surface. On evaluating implant survival according to texture, Lee et al. (24) found the platform insertion depth to be a decisive factor. The failure rate of the hydroxyapatite-coated Bicon® implants positioned at crestal level and 2 mm subcrestal was 10.29% and 3.01%, respectively, after a mean follow-up of 3.3 years. The authors concluded that bacterial contamination is more likely with rough-surfaced implants after the prosthetic phase when the platform is positioned at crestal level. In this context, Todescan et al. (25) postulated that platforms placed at subcrestal level tend to extend the mucosal barrier, and the probability of periimplantitis after the prosthetic phase is therefore lower in the case of implants placed 2 mm below the bone crest than in implants positioned at crestal level.

A number of studies have used a range of commercial platform switching systems such as Prevail (Biomet 3i)® (16,20,23,27), Osseotite Certain (Biomet 3i)® (8,10), Global (Sweden & Martina)® (2,15), Novel Active (Nobel Biocare)® (19), or Morse taper-type connections such as Ankylos (Dentsply Friadent)® (15). In other studies, platform reduction was achieved by using a prosthetic abutment of lesser diameter than the diameter of the implant platform, thereby creating controlled horizontal implant-abutment disadjustment (10). According to Cochran et al. (14), the Morse taper-type internal connection results in lesser bone loss than the butt-joint connection, because it avoids bacterial contamination and is therefore associated with a lesser inflammatory infiltrate at the implant-abutment interface. The drawing of conclusions becomes more complicated when we moreover also consider the magnitude of horizontal disadjustment. The existence of abutments of several diameters means that they can be used indistinctly to produce such disadjustment. Becker et al. (28) recorded differences in marginal bone loss possibly attributable to the different horizontal disadjustment magnitudes created (0.3 mm versus 0.5 mm). When disadjustment was 0.3 mm and 0.5 mm, the mean marginal bone loss was seen to be 1.2±0.2 mm and 1.3±0.4 mm, respectively, one month after surgery.

The surgical technique in one or two steps is also a confounding factor to be taken into account. With the single-step technique or after second surgery, the implant-abutment interface is established, and bacterial contamination and inflammatory infiltration of the interface occurs. The unintended exposure of submerged implants during the healing period can result in early bone loss (2,29-30). This does not occur while the implants remain submerged. However, Jung et al. (18) found no significant differences in terms of bone loss between submerged and non-submerged implants.

The initial soft tissue thickness (keratinized gums) can intervene in marginal bone reabsorption in implants placed at supracrestal level. In a pilot study, Linkevicius et al. (16) found that horizontal disadjustment in platform switching does not prevent bone reabsorption if the mucosal thickness at the time of implant placement is 2 mm or less.

Other parameters such as the type of connection (internal/external)(14), or the inclination of the implant, could also influence marginal bone loss. The radiographic measurement techniques differed among the examined studies. As coronal reference points, most authors used the implant-abutment interface, with the first bone-implant contact point (fBIC) as the most apical point (10,17). The coronal reference point used by Vigolo et al. (11) was the most apical point of the smooth neck of the implant, inserted at crestal level. This lack of consensus in the measurements gives rise to differences in estimating the millimeters of marginal bone loss.

Implant survival rate

The survival rates of the platform switching implants ranged from 88.6% (19) to 100% (2,8,12,16,23). In relation to the platform insertion depth, the survival rate of the implants placed at crestal level was 88.6% (19) to 100% (2,16), versus 98.3% (7) to 100% below crestal level (8,15,23), and 100% above crestal level (13). Veis et al. (10) found healing of the hard and soft peri-implant tissues in the platform switching implants to be successful, independently of the location of the implant neck and the consequent degree of marginal bone reabsorption.

## Conclusions

Platform switching and platform insertion depth are two independent factors in relation to marginal bone reabsorption. In turn, synergic effects may be observed between them: by increasing the horizontal and vertical distance between the implant-abutment interface and the marginal bone crest, the inflammatory infiltrate is displaced away from the marginal crestal bone, with a reduction in bone loss. In the implants positioned at supracrestal level, bone loss was slightly less pronounced than in the case of those positioned at subcrestal level – though statistical significance was not reached. Nevertheless, crestal insertion or positioning 1-2 mm subcrestal has been recommended. Differences in the results of several studies, and the existence of confounding factors, explains the lack of agreement among authors regarding the ideal insertion depth.
